# Supplementary material for: Chromothripsis during telomere crisis is independent of NHEJ, and consistent with a replicative origin
Source: Genome Res. 2019 May;29(5):737–49. doi: 10.1101/gr.240705.118 (PMC6499312; doi:10.1101/gr.240705.118)
Supplement: Supplemental Material [file supp_gr.240705.118_Supplemental_file_1.zip › contigs/annotated_contigs/DB53/contig.2.DB53_length_427_mean_cov_5.26932084309.docx]

**DB53_length_427_mean_cov_5.26932084309**

```
AGTGAAAAATCTCCTTCCTCCCTGGGCCCCATTCACCCAGTTCTTACACCACTCTAATAGGAAACTATTATTTTGGGTTCTATGTACAT
 >chr4:19079095-19079465 + E=1e-211 p=0e+00
TCTCACAGAGCTGTTAGGCAGAAAAAAGCAATGCATAATGCTTGTTTGTTCAATTTTTGACAACTTTGTTAAGGTATAATTGGCATACA

ATACATGATACATGTTTAACATGTATAATTTCATAATTTCTGACATAAATACGTCCATGAAACCCTCCCACAATCAAGAGAGTAAACAT

ATCCATCAATCTCAAAAGCTTCCTTGCACTCCTTTGTCATCTCTCTCTCCTGCCCTCCCATGTCACTCCCACCTCCATCCCCAAGCAA|

CCACTGATTTGCTTT|ATGTTACTGTAGATTAGTTTATATTTTCCAGATACACTGGTTTATTTTACTCAGCAT>chr4:19085550-19085622 + E=8e-32
```
